# Emerging *Corynebacterium diphtheriae* Species Complex Infections, Réunion Island, France, 2015–2020

**DOI:** 10.3201/eid2908.230106

**Published:** 2023-08

**Authors:** Thomas Garrigos, Anais Grimal, Edgar Badell, Nicolas Traversier, Sandrine Picot, Anne Lignereux, Mahery Ramiandrisoa, Céline Ben Cimon, Marie-Christine Jaffar-Bandjee, Houssein Gbaguidi-Haore, Julie Toubiana, Sylvain Brisse, Guillaume Miltgen, Olivier Belmonte

**Affiliations:** Félix Guyon University Hospital Center, Saint-Denis, Réunion Island, France (T. Garrigos, A. Grimal, N. Traversier, M.-C. Jaffar-Bandjee, G. Miltgen, O. Belmonte);; Processus Infectieux en Milieu Insulaire Tropical, University of La Réunion, Inserm, CNRS, IRD, Saint-Denis, Réunion Island, France (T. Garrigos, G. Miltgen);; Institut Pasteur University of Paris Cité, Paris, France (E. Badell, J. Toubiana, S. Brisse);; Institut Pasteur National Reference Center for Corynebacteria of the Diphtheriae Complex, Paris (E. Badell, J. Toubiana, S. Brisse);; University Hospital of La Réunion, Saint Pierre, Réunion Island, France (S. Picot);; Hospital Center West Réunion, Saint Paul, Réunion Island, France (A. Lignereux);; Cerballiance Laboratory, Le Port, Réunion Island, France (M. Ramiandrisoa);; Inovie RéuniLAB Laboratory, Sainte-Clotilde (C. Ben Cimon);; University Hospital Center and University of Bourgogne Franche-Comté, Besançon, France (H. Gbaguidi-Haore);; Necker-Enfants malades Hospital, Paris (J. Toubiana).

**Keywords:** diphtheria, *Corynebacterium diphtheriae*, *Corynebacterium ulcerans*, epidemiology, genotyping, antimicrobial resistance, bacteria, respiratory infections, zoonoses, Réunion Island, France

## Abstract

Clinical, epidemiologic, and microbiologic analyses revealed emergence of 26 cases of *Corynebacterium diphtheriae* species complex infections on Réunion Island, France, during 2015–2020. Isolates were genetically diverse, indicating circulation and local transmission of several diphtheria sublineages. Clinicians should remain aware of the risk for diphtheria and improve diagnostic methods and patient management.

Diphtheria is a contagious, potentially fatal infection caused by toxin-producing bacteria of the *Corynebacterium* *diphtheriae* species complex, which includes *C*. *diphtheriae*, *C*. *ulcerans*, *C*. *pseudotuberculosis*, *C*. *rouxii*, *C*. *belfantii*, and *C*. *silvaticum*. Infection is localized principally in the upper respiratory tract, and production of diphtheria toxin (encoded by the *tox* gene) can cause systemic complications. Cutaneous diphtheria and diphtheria endocarditis can also act as sources of respiratory infections (*1*–*4*). Diphtheria surveillance has traditionally focused on respiratory illness caused by toxigenic *C*. *diphtheriae* but has been expanded in some countries to include all *C*. *diphtheriae* species complex infections irrespective of species, infection site, or toxigenicity, enabling broader disease monitoring. *C*. *diphtheriae* spreads via human-to-human contact; *C*. *ulcerans* and *C*. *pseudotuberculosis* are transmitted to humans primarily through animal contact.

Diphtheria was once a major cause of infant death, but global incidence has declined over the past century, largely because of mass vaccination. Consequently, diphtheria is now often considered a forgotten disease (*5*). Nevertheless, diphtheria reemergence has been reported in high-income countries and is closely related to patient travel history. Diphtheria is considered endemic in Madagascar, Comoros, and Mayotte in the southwest Indian Ocean, but few cases have been reported on other islands, including Réunion Island, an overseas department of France, where cases emerged in 2015 (*6*,*7*). Vaccination coverage is poorer in Mayotte (45% for 7- to 11-year-old children) than in Réunion Island (96% for children 11 months of age). Recent improvements in laboratory diagnostic capabilities, such as mass spectrometry use, have increased reports of *C*. *diphtheriae* species complex infections (*8*). However, knowledge of prevalence and origin of those infections is limited in this region. The aims of this study were to review the clinical, epidemiologic, and microbiologic characteristics of *C*. *diphtheriae* species complex infections on Réunion Island during 2015–2020 and identify possible links with cases on other islands in the region.

## The Study

We included all cases of *C*. *diphtheriae* species complex infections reported to the regional health agency and recorded at Réunion Island University Hospital during 2015–2020. We analyzed medical records and extracted age, sex, country of residence, recent travel, contact with animals, socioeconomic status, and diphtheria vaccination status for each case. We performed antimicrobial susceptibility testing; identified co-infecting strains; and determined *tox* gene presence, diphtheria toxin production, and biovar and sequence type (ST). We sent each isolate to the National Reference Center for Corynebacteria of the *diphtheriae* Complex (Institut Pasteur, Paris, France) to confirm species identity through multiplex PCR and biotyping as previously described (*8*–*10*). We detected the *tox* gene by using conventional PCR or, since 2019, by using multiplex real-time PCR (*10*). We assessed toxin production by using a modified Elek test (*11*). We determined antimicrobial drug susceptibility by using disk diffusion or by determining MICs (E-test; bioMérieux, https://www.biomerieux.com), in accordance with CASFM/EUCAST2021 (https://www.sfm-microbiologie.org/2021/04/23/casfm-avril-2021-v1-0) recommendations for benzylpenicillin, amoxicillin, cefotaxime, clindamycin, rifampin, and ciprofloxacin. We genotyped each isolate by using multilocus sequence typing (MLST) (*12*).

A total of 26 cases of *C*. *diphtheriae* species complex infections were recorded, from which 27 *C*. *diphtheriae* and 2 *C*. *ulcerans* isolates were cultured. Most (88.5%) infected patients were male; median age was 60 (interquartile range 32.5–67) years. Fourteen (50%) patients lived on Réunion Island, 3 (11.5%) in Mayotte, 4 (19.2%) in mainland France, 3 (11.5%) in Comoros, and 2 (7.8%) in Madagascar. Most (84.6%) patients had skin manifestations, and 16 patients were vaccinated (Table 1; Appendix Figure). Of 24 *C*. *diphtheriae* infections, 8 occurred in patients who had recently traveled to or originated from Madagascar, 4 who traveled to or originated from Mayotte, and 3 who traveled to or originated from Comoros. Since 2018, a total of 9 cases on Réunion Island have been considered locally acquired; all of those patients lived in poor socioeconomic conditions. *C*. *ulcerans* infections occurred in 2 patients living on Réunion Island who had not traveled recently but had contact with animals (Table 1; Figure). We performed a Spearman rank correlation to compare locally acquired strains isolated during 2015–2018 and 2019–2020; a 75% increase in locally acquired *C*. *diphtheriae* infections occurred in 2019–2020 (ρ = 0.8452; p = 0.0341).

**Table 1:** Demographic and clinical features of 26 patients in study of emerging *Corynebacterium diphtheriae* species complex infections, Réunion Island, France, 2015–2020*

Patient no.	Isolates	Year	Age, y/Sex	Animal contact	Country	Travel	Immunized	Comorbidities/living conditions	Symptoms
1	CD1/FRC0304	2015	9/M	None	Mayotte, France	No	Yes	Disadvantaged priority district	Bullous pemphigoid
	CD2/FRC0316	2015							Pneumonia
2	CD3/FRC0314	2015	71/M	None	Mainland France	Madagascar	No	Diabetic	Left lower limb ulceration
3	CD4/FRC0376	2015	13/M	None	Mayotte, France	No	Yes	Disadvantaged district, autoimmune hepatitis	Left lower limb ulceration
4	CD5/FRC0383	2016	78/M	None	Réunion Island	Madagascar	No	Diabetic, heart failure, COPD	Right lower limb ulceration
	CD6/FRC0393	2016							Phlyctenule, right leg
5	CD7/FRC0385	2016	64/M	None	Mainland France	Madagascar	No	High blood pressure	Left lower limb ulceration
6	CU1/FRC0391	2016	67/F	Dogs, cats	Réunion Island	No	Unknown	Alcoholism, obesity, high blood pressure	Left lower limb ulceration
7	CD8/FRC0402	2016	67/M	None	Réunion Island	Madagascar	Yes	High blood pressure	Left lower limb ulceration
8	CD9/FRC0410	2016	54/M	None	Réunion Island	Madagascar	Unknown	Unspecified	Ankle wound
9	CD10/FRC0423	2016	5/F	None	Comoros	No	Yes	Disadvantaged district	Lower limb ulceration
10	CD11/FRC0477	2017	74/M	None	Madagascar	No	Yes	Unspecified	Ulceration of right toe
11	CD12/FRC0501	2017	66/M	None	Madagascar	No	No	Diabetic, COPD	Left lower limb ulceration
12	CD13/FRC0624	2018	60/M	None	Mayotte (France)	No	No	Unspecified	Ulceration of right toe
13	CD14/FRC0630	2018	68/M	None	Mainland France	Madagascar	Yes	Diabetic, heart failure	Wound, right tibia
14	CD15/FRC0733	2019	2/M	None	Comoros	No	Yes	Disadvantaged district	Ulceration of right toe
15	CD16/FRC0782	2019	38/M	None	Réunion Island	No	Yes	Homelessness, alcoholism	Lower limb ulceration
	CD17/FRC0809	2019							
16	CD18/FRC0819	2019	27/M	None	Réunion Island	Mayotte	No	Alcoholism, hepatitis B	Right ankle ulceration
17	CU2/FRC0820	2019	76/M	Dogs	Réunion Island	No	Unknown	Alcoholism, diabetic	Metatarsus damage, left foot
18	CD19/FRC0849	2019	39/F	None	Comoros	No	Yes	Lupus	Lower limb ulceration
19	CD20/FRC0865	2020	59/M	None	Réunion Island	No	Unknown	Unspecified	Lower limb ulceration
20	CD21/FRC0875	2020	55/M	None	Réunion Island	No	Unknown	Unspecified	Left malleolar ulceration
21	CD22/FRC0893	2020	17/M	None	Mainland France	No	Unknown	Unspecified	Foot ulceration
22	CD23/FRC0928	2020	33/M	None	Réunion Island	No	Unknown	Homelessness, alcoholism	Foot ulceration
23	CD24/FRC0970	2020	59/M	None	Réunion Island	No	Yes	Homelessness, alcoholism	Elbow abscess
24	CD25/FRC0975	2020	32/M	None	Réunion Island	No	Yes	Homelessness, alcoholism	Ulcerations of feet
25	CD26/FRC1050	2020	53/M	None	Réunion Island	No	Yes	Homelessness, alcoholism	Metatarsus damage, left foot
26	CD27/FRC1065	2020	67/M	None	Réunion Island	No	Yes	High blood pressure, frontal squamous cell carcinoma	Frontal sinus damage

*CD, *Corynebacterium diphtheriae*; COPD, chronic obstructive pulmonary disease; CU, C. *ulcerans*.

**Figure:**
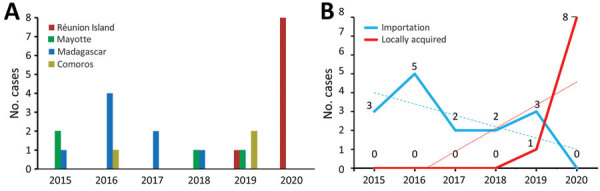
Number of cases diagnosed per year in study of emerging *Corynebacterium diphtheriae* species complex infections, Réunion Island, France, 2015–2020. Number of cases were classified according to geographic origin (A) or travel history of patients (B). Dotted lines indicate linear trends.

Isolates were obtained from cutaneous lesion (n = 24), bone (n = 4), and respiratory (n = 1) samples. Eight of 27 *C*. *diphtheriae* isolates were toxigenic, yielding positive Elek test results. The 2 *C*. *ulcerans* isolates were nontoxigenic. *C. diphtheriae* isolates were characterized as biovars Mitis (n = 20) and Gravis (n = 7).

Patient isolates were co-infected most frequently with *Staphylococcus* *aureus* (n = 17) and *Streptococcus* *pyogenes* (n = 18). Benzylpenicillin resistance was observed in 80% of isolates according to CASFM/EUCAST2021 recommendations, but isolates were categorized as susceptible increased exposure according to EUCAST version 13.0 proposed breakpoints (https://www.eucast.org/clinical_breakpoints) (Appendix Table). One (3.5%) *C*. *diphtheriae* isolate was resistant to amoxicillin (CD8/FRC0402; MIC 1.5 mg/L), and 1 was resistant to rifampin. Both *C*. *ulcerans* isolates were resistant to clindamycin (100%, natural low susceptibility), whereas clindamycin resistance was observed for only 1 *C*. *diphtheriae* isolate.

We identified 21 STs by MLST analysis, including ST88 for *C*. *diphtheriae* isolates from 4 patients and ST339 for both *C*. *ulcerans* isolates (Table 2). All *C*. *diphtheriae* STs had 2–5 mismatches, except ST87 and ST237, which had 1 mismatch between them. ST339 (*C*. *ulcerans*) had 7 mismatches with all *C*. *diphtheriae* STs.

**Table 2:** Characteristics of isolates from 26 patients in study of emerging *Corynebacterium diphtheriae* species complex infections, Réunion Island, France, 2015–2020*

Patient no.	Isolate	Year	Isolation site	Species	Biovar	*tox* gene	Elek test	ST†	Co-infections‡
1	CD1/FRC0304	2015	Cutaneous	*C. diphtheriae*	Gravis	Negative	NA	102	*S. pyogenes, S. aureus, A. haemolyticum*
	CD2/FRC0316	2015	Respiratory	*C. diphtheriae*	Mitis	Negative	NA	95	*S. aureus*
2	CD3/FRC0314	2015	Cutaneous	*C. diphtheriae*	Mitis	Positive	Positive	421	*S. aureus*
3	CD4/FRC0376	2015	Cutaneous	*C. diphtheriae*	Gravis	Positive	Positive	388	*S. pyogenes*
4	CD5/FRC0383	2016	Cutaneous	*C. diphtheriae*	Mitis	Negative	NA	423	*S. pyogenes, S. aureus*
	CD6/FRC0393	2016	Cutaneous	*C. diphtheriae*	Mitis	Negative	NA	423	*S. pyogenes, S. aureus*
5	CD7/FRC0385	2016	Cutaneous	*C. diphtheriae*	Mitis	Positive	Positive	91	*S. pyogenes*
6	CU1/FRC0391	2016	Cutaneous	*C. ulcerans*	NA	Negative	NA	**339**	*S. dysgalactiae*
7	CD8/FRC0402	2016	Cutaneous	*C. diphtheriae*	Mitis	Negative	NA	410	*S. dysgalactiae*
8	CD9/FRC0410	2016	Cutaneous	*C. diphtheriae*	Mitis	Negative	NA	415	*S. pyogenes*
9	CD10/FRC0423	2016	Cutaneous	*C. diphtheriae*	Gravis	Negative	NA	101	*S. aureus*
10	CD11/FRC0477	2017	Cutaneous	*C. diphtheriae*	Gravis	Negative	NA	481	*S. pyogenes, S. aureus, A. haemolyticum*
11	CD12/FRC0501	2017	Cutaneous	*C. diphtheriae*	Gravis	Positive	Positive	521	*S. pyogenes*
12	CD13/FRC0624	2018	Bone	*C. diphtheriae*	Mitis	Negative	NA	237	*S. aureus*
13	CD14/FRC0630	2018	Cutaneous	*C. diphtheriae*	Gravis	Negative	NA	606	*S. pyogenes, S. aureus*
14	CD15/FRC0733	2019	Cutaneous	*C. diphtheriae*	Mitis	Negative	NA	351	*S. pyogenes*
15	CD16/FRC0782	2019	Cutaneous	*C. diphtheriae*	Mitis	Positive	Positive	688	*S. pyogenes, S. aureus*
	CD17/FRC0809	2019	Cutaneous	*C. diphtheriae*	Mitis	Positive	Positive	688	*S. aureus*
16	CD18/FRC0819	2019	Cutaneous	*C. diphtheriae*	Gravis	Positive	Positive	87	*S. pyogenes, A. haemolyticum*
17	CU2/FRC0820	2019	Bone	*C. ulcerans*	NA	Negative	NA	**339**	*S. aureus*
18	CD19/FRC0849	2019	Cutaneous	*C. diphtheriae*	Mitis	Positive	Positive	426	*S. pyogenes, S. aureus*
19	CD20/FRC0865	2020	Cutaneous	*C. diphtheriae*	Mitis	Negative	NA	102	*S. pyogenes, S. aureus*
20	CD21/FRC0875	2020	Cutaneous	*C. diphtheriae*	Mitis	Negative	NA	707	*S. pyogenes*
21	CD22/FRC0893	2020	Cutaneous	*C. diphtheriae*	Mitis	Negative	NA	708	*S. pyogenes*
22	CD23/FRC0928	2020	Cutaneous	*C. diphtheriae*	Mitis	Negative	NA	**88**	*S. pyogenes*
23	CD24/FRC0970	2020	Cutaneous	*C. diphtheriae*	Mitis	Negative	NA	**88**	*S. pyogenes, S. aureus, A. haemolyticum*
24	CD25/FRC0975	2020	Cutaneous	*C. diphtheriae*	Mitis	Negative	NA	**88**	*S. aureus*
25	CD26/FRC1050	2020	Bone	*C. diphtheriae*	Mitis	Negative	NA	771	*A. haemolyticum*
26	CD27/FRC1065	2020	Bone	*C. diphtheriae*	Mitis	Negative	NA	**88**	*S. aureus*

*CD, *Corynebacterium diphtheriae*; CU, C. *ulcerans*; NA, not applicable; ST, sequence type. †Numbers in bold indicate a common ST shared among strains from different patients. ‡Co-infections with *Arcanobacterium* *haemolyticum*, *Staphylococcus* *aureus*, *Streptococcus* *dysgalactiae*, or *Streptococcus* *pyogenes*.

## Conclusions

We report increased prevalence of cutaneous *C*. *diphtheriae* species complex infections on Réunion Island during 2015–2020. Introduction of mass spectrometry analysis in hospital laboratories and increased clinician awareness might have led to increased case reporting. Our study confirms that *C*. *diphtheriae* species complex members are circulating and are likely underestimated in the southwest Indian Ocean (*7*,*13*). Moreover, we observed emergence of locally acquired cutaneous *C*. *diphtheriae* infections on Réunion Island since 2019. The number of imported cases in 2020 was probably limited because of the COVID-19 pandemic, which reduced travel. Indeed, all *C*. *diphtheriae* cases identified during 2015–2018 occurred in patients who had traveled from other islands in the Indian Ocean. In addition, cutaneous diphtheria appeared to be associated with poor socioeconomic living conditions, in which alcoholism, drug dependence, and homelessness are factors that increase risk for human-to-human transmission and virulence (*14*).

A total of 8 (30%) *C*. *diphtheriae* isolates were toxigenic and caused cutaneous infections. Nontoxigenic *C*. *diphtheriae* isolates (70%, n = 19) were obtained from cutaneous lesions, respiratory samples, and bone samples. Clinicians should be aware that nontoxigenic *C*. *diphtheriae* can potentially cause severe disease (*1*,*14*,*15*). Moreover, all isolates were co-infected with pyogenic bacteria, suggesting diphtheria infection should be considered under polymicrobial conditions.

MLST analysis identified 21 different STs; most were unrelated (>2 mistmatches) reflecting marked genetic diversity of isolates. ST88 was found in 4 patients living on Réunion Island who had not traveled recently, indicating probable local acquisition. ST88 had previously been reported only in patients from Mayotte. Therefore, our results show that multiple *C*. *diphtheriae* species complex clones are circulating in the southwest Indian Ocean (*8*). Both *C*. *ulcerans* strains belonged to ST339. The National Reference Center reported that ST339 is the predominant *C*. *ulcerans* ST found in animals in France. Although considerable ST diversity was revealed, whole-genome sequencing will be required to further evaluate circulating *C*. *diphtheriae* clones in this region.

In conclusion, we describe emergence of locally acquired *C. diphtheriae* species complex infections on Réunion Island during 2019–2020. Local clinicians and microbiologists should remain aware of this neglected infection; improvements should be made in diagnostic methods and management of infected patients, such as maintaining availability of diphtheria antitoxin.

Appendix Additional information for emerging *Corynebacterium* *diphtheriae* species complex infections, Réunion Island, France, 2015–2020.
