# Supplementary material for: Emerging Corynebacterium diphtheriae Species Complex Infections, Réunion Island, France, 2015–2020
Source: Emerg Infect Dis. 2023 Aug;29(8):1630–3. doi: 10.3201/eid2908.230106 (PMC10370861; doi:10.3201/eid2908.230106)
Supplement: Appendix — Additional information for emerging Corynebacterium diphtheriae species complex infections, Réunion Island, France, 2015–2020. [file 23-0106-Techapp-s1.pdf]

Article DOI: https://doi.org/10.3201/eid2908.230106
EID cannot ensure accessibility for supplementary materials supplied by authors. Readers who have difficulty accessing supplementary content should contact the authors for assistance.

Emerging *Corynebacterium diphtheriae* Species Complex Infections, Réunion Island, France, 2015–2020

Appendix

Appendix Table. Sensitivity of different patient isolates to antimicrobial drugs*

Isolates	Benzylpenicillin	Amoxicillin	Clindamycin	Rifampin	Ciprofloxacin
CD1/FRC0304	SIE	S	S	S	S
CD2/FRC0316	SIE	S	S	S	S
CD3/FRC0314	SIE	S	S	S	S
CD4/FRC0376	SIE	S	S	S	S
CD5/FRC0383	SIE	S	S	S	S
CD6FRC0393	SIE	S	S	S	S
CD7/FRC0385	SIE	S	S	S	S
CU1/FRC0391	SIE	S	R	S	S
CD8/FRC0402	SIE	R	R	S	S
CD9/FRC0410	SIE	S	S	S	S
CD10/FRC0423	SIE	S	S	S	S
CD11/FRC0477	SIE	S	S	S	S
CD12/FRC0501	SIE	S	S	S	S
CD13/FRC0624	SIE	S	S	S	S
CD14/FRC0630	SIE	S	S	R	S
CD15/FRC0733	SIE	S	S	S	S
CD16/FRC0782	SIE	S	S	S	S
CD17/FRC0809	SIE	S	S	S	S
CD18/FRC0819	SIE	S	S	S	S
CU2/FRC0820	SIE	S	R	S	S
CD19/FRC0849	SIE	S	S	S	S
CD20/FRC0865	SIE	S	S	S	S
CD21/FRC0875	SIE	S	S	S	R
CD22/FRC0893	SIE	S	S	S	S
CD23/FRC0928	SIE	S	S	S	S
CD24/FRC0970	SIE	S	S	S	S
CD25/FRC0975	SIE	S	S	S	S
CD26FRC1050	SIE	S	S	S	S
CD27/FRC1065	SIE	S	S	S	S

*CD, *Corynebacterium diphtheriae*; CU, C. *ulcerans*; R, resistant; S, sensitive; SIE, susceptible increased exposure.

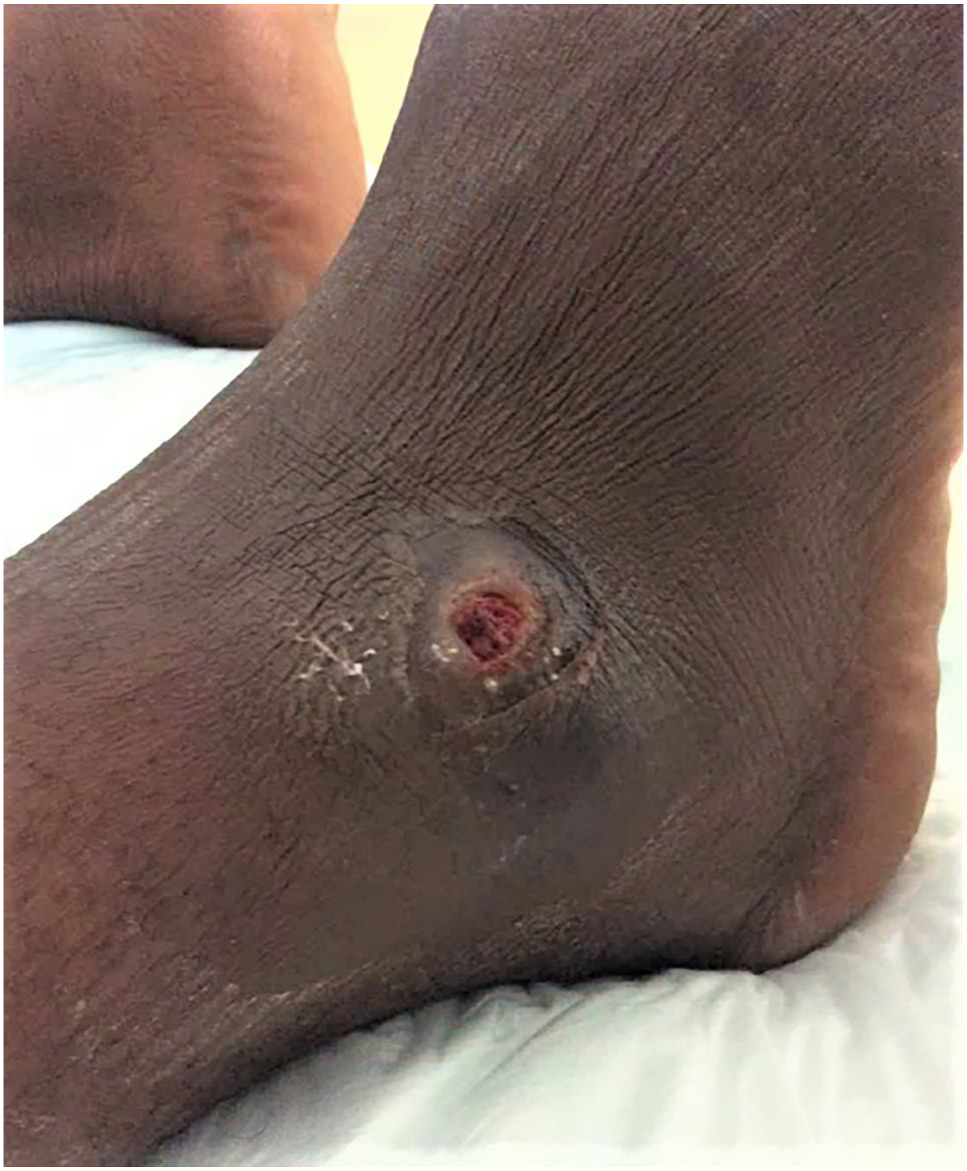

Appendix Figure. Image of right ankle cutaneous ulcer from patient with *Corynebacterium diphtheriae* species complex infection, Réunion Island, France, 2015–2020.
